# Prognostic factors affecting postoperative survival of patients with solitary small hepatocellular carcinoma

**DOI:** 10.1186/s40880-016-0143-x

**Published:** 2016-08-16

**Authors:** Mu-Yan Cai, Feng-Wei Wang, Chang-Peng Li, Li-Xu Yan, Jie-Wei Chen, Rong-Zhen Luo, Jing-Ping Yun, Yi-Xin Zeng, Dan Xie

**Affiliations:** 1 State Key Laboratory of Oncology in South China, Department of Pathology, Collaborative Innovation Center for Cancer Medicine, Sun Yat-sen University Cancer Center, No. 651, Dongfeng Road East, Guangzhou, 510060 Guangdong P. R. China; 2 Department of Pathology and Laboratory Medicine, Guangdong Provincial People’s Hospital, Guangzhou, 510080 Guangdong P. R. China

**Keywords:** Small hepatocellular carcinoma, Tumor size, Vascular invasion, Prognosis

## Abstract

**Background:**

Small hepatocellular carcinoma (sHCC) is a unique variant of HCC that is characterized by small tumor size (maximum tumor diameter ≤3 cm) and favorable long-term outcomes. The present study aimed to define clinicopathologic factors that predict survival in patients with sHCC.

**Methods:**

The study population consisted of 335 patients who underwent hepatectomy for solitary sHCC between December 1998 and 2010. Prognostic factors were evaluated using Kaplan–Meier curves and Cox proportional hazard models.

**Results:**

The 5-year overall survival (OS) and recurrence-free survival (RFS) rates were 77.7% and 59.9%, respectively. Kaplan–Meier curves showed that tumor size and vascular invasion had prognostic significance within this relatively selected cohort (*P* < 0.05). Multivariate analysis confirmed that increased tumor size and vascular invasion were independent prognostic factors for short OS (hazard ratio [HR] = 2.367, 95% confidence interval [CI] 1.406–3.985; HR = 2.954, 95% CI 1.781–4.900) and RFS (HR = 1.779, 95% CI 1.259–2.514; HR = 1.699, 95% CI 1.165–2.477) in sHCC patients (*P* < 0.05). Importantly, a proposed prognostic scoring model was derived according to the two variables; tumor size and extent of vascular invasion were significantly associated with OS and RFS in patients with sHCC (*P* < 0.001).

**Conclusions:**

Tumor size and vascular invasion are feasible and useful prognostic factors for sHCC. The proposed prognostic model, based on tumor size and vascular invasion, is informative in predicting survival in sHCC patients undergoing hepatectomy.

## Background

Hepatocellular carcinoma (HCC) is the third leading cause of cancer-related deaths worldwide [1]. HCC has a high prevalence in Southeast Asia and Africa [2], and the incidence has been steadily increasing in both Europe and the United States [3]. Liver cirrhosis, particularly as a consequence of hepatitis B virus (HBV) and hepatitis C virus (HCV) infection, is the most common risk factor for the development of HCC [4]. Due to the high prevalence of HBV infection among Chinese populations, there are a significant number of deaths due to HBV-related HCC in China [5]. Surveillance and recent advances in imaging techniques have resulted in improved identification and diagnosis of small HCC (sHCC, ≤3 cm in diameter) [6, 7].

Early detection of HCC allows for curative or palliative treatment with surgical resection or transcatheter arterial chemoembolization [8]. Currently, surgical resection offers the greatest chance for cure; however, many patients still suffer from disease recurrence after primary treatment [9, 10]. It is imperative to identify patients at high risk for optimal follow-up or postoperative adjuvant therapies in these patients [11, 12]. The known risk factors for sHCC enable the identification and screening of patients at high risk for HCC; however, prognosis-related factors are yet to know, which would enable stratification of sHCC patients into treatment groups following hepatectomy [13–15]. Clinicopathologic features are the main factors affecting postoperative survival of patients with sHCC. The current study was to identify clinicopathologic factors that predict survival of sHCC patients who underwent hepatectomy.

## Patients and methods

### Patients

Data were obtained from the Sun Yat-sen University Cancer Center (Guangzhou, China) for cases of pathologically confirmed, non-metastatic sHCC between December 1998 and 2010. Only patients who underwent surgical resection, not ablation or transplantation, as the first course of therapy were included in the present study. Case data were collected retrospectively using the following eligibility criteria: (1) solitary sHCC (≤3 cm) only, (2) presence of the HBV surface antigen, (3) patient underwent primary and curative resection, (4) no evidence of metastatic or residual disease, (5) no preoperative adjuvant therapy, and (6) availability of complete follow-up data. Patients with unknown cause of death were excluded. All patients underwent curative resection for HCC with the following intraoperative goals: a resection margin >1 cm, complete tumor resection, and leaving the resection margin free of tumor. All the patients included in our study were previously diagnosed by senior pathologists, and the histopathologic diagnosis was re-confirmed independently by an experienced pathologist (Mu-Yan Cai). The extent of tumor differentiation was determined based on the criteria proposed by Edmonson and Steiner [16]. Vascular invasion in each HCC specimen was identified in several serial cross sections. Patients who had macroscopic and/or microscopic tumor emboli within the large capsular vessels, the central hepatic vein, or the portal vein were considered to have vascular invasion. Cirrhosis was defined by the presence of fibrous septa throughout the liver that subdivided the parenchyma into nodules. The Institutional Research Medical Ethics Committee of the Sun Yat-sen University Cancer Center granted approval for this study. No informed consent (written or verbal) was obtained for the use of retrospective tissue samples from the patients in this study, most of whom were deceased. Consent was not deemed necessary by the Ethics Committee, who waived the need for consent, however all samples were anonymized.

### Follow-up

After partial hepatectomy, patient follow-up was performed at 2–6 month intervals at the outpatient clinic with regular surveillance for recurrence using serum alpha-fetoprotein (AFP) examination, abdominal ultrasonography, and chest radiography. In cases where tumor recurrence or metastasis was suspected, further examinations, including computed tomography (CT) and magnetic resonance imaging (MRI), were performed. Biopsies were obtained when necessary. Recurrence-free survival (RFS) was calculated from the date of surgery to the first documentation of cancer recurrence. Cancer-specific overall survival (OS) was calculated from the date of surgery to the date of the last follow-up visit (January 18, 2014) or time of death attributed to HCC.

### Statistical analysis

Kaplan–Meier analysis with a log-rank test was performed to compare the survival distributions of different variables. Multiple Cox proportional hazard regression was performed to identify the independent factors that had a significant impact on patient survival. All statistical analyses were conducted using SPSS for Windows version 13.0 release (SPCC Inc., Chicago, IL, USA). A difference was considered significant if the *P* value from a two-tailed test was less than 0.05.

## Results

### Patient characteristics

Using the criteria described above, 335 cases of sHCC were included in this study. Demographic and clinical findings for these patients are presented in Table 1. Of the 335 patients, 295 (88.1%) were men, and 40 (11.9%) were women, with a median age of 48 years (range, 26–78 years). Among the 335 patients, 196 (58.5%) had serum AFP levels >20 ng/mL. The median tumor size was 2.5 cm (range, 0.6–3.0 cm). Most patients exhibited well-differentiated or moderately-differentiated tumors (*n* = 264, 78.8%). Vascular invasion was observed in 80 (23.9%) patients, characterized by clusters of tumor cells localized to vascular spaces, and interspersed with blood cells. A total of 121 (36.1%) tumors were encapsulated. Liver cirrhosis was present in 134 (40.0%) patients. Patients with confirmed tumor recurrence underwent re-resection when possible, or were treated with transcatheter arterial chemoembolization, percutaneous ethanol injection, or radiofrequency ablation.

**Table 1:** The characteristics of 335 patients with small hepatocellular carcinoma (sHCC)

Variable	No. of patients (%)
Gender	
Male	295 (88.1)
Female	40 (11.9)
Age (years)	
≤48	166 (49.6)
>48	169 (50.4)
ALT (U/L)	
≤40	192 (57.3)
>40	143 (42.7)
AFP (ng/mL)	
≤20	139 (41.5)
>20	196 (58.5)
Tumor size (cm)	
≤2.5	188 (56.1)
>2.5	147 (43.9)
Tumor differentiation	
Well	56 (16.7)
Moderate	208 (62.1)
Poor	63 (18.8)
Undifferentiated	8 (2.4)
Vascular invasion	
Absent	255 (76.1)
Present	80 (23.9)
Envelope	
Absent	214 (63.9)
Present	121 (36.1)
Liver cirrhosis	
Absent	201 (60.0)
Present	134 (40.0)
Recurrence	
Absent	204 (60.9)
Present	131 (39.1)

*ALT* alanine aminotranferease, *AFP* alpha-fetoprotein

### Prognostic factors affecting postoperative survival of patients with sHCC

**Fig. 1:**
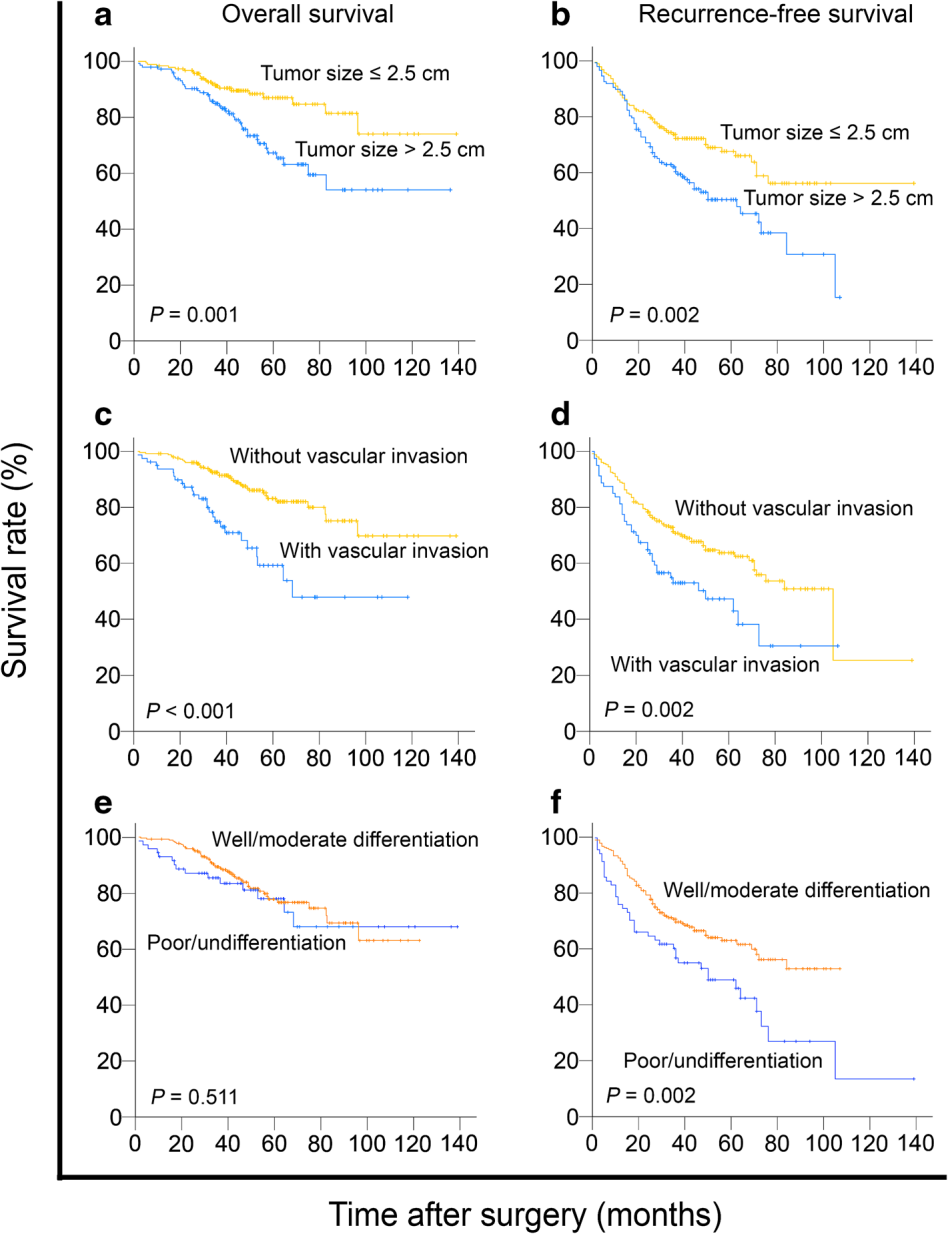
Prognostic factors affecting postoperative survival of patients with small hepatocellular carcinoma (sHCC) (log-rank test). **a** Tumor size >2.5 cm was associated with a decrease in overall survival (OS) of patients (*P* = 0.001). **b** Tumor size >2.5 cm was associated with a decrease in recurrence-free survival (RFS) of patients (*P* = 0.002). **c** Tumor with vascular invasion was associated with a decrease in OS of patients (*P* < 0.001). **d** Tumor with vascular invasion was associated with a decrease in RFS of patients (*P* = 0.002). **e** Tumor with poor/undifferentiation was not significantly associated with a decrease in OS of patients (*P* = 0.511). **f** Tumor with poor/undifferentiation was associated with a decrease in RFS of patients (*P* = 0.002)

Of the 335 patients, 131 (39.1%) experienced HCC recurrence, and 62 (18.5%) died during the follow-up period. The median OS and RFS were 44 and 36 months. The 5-year OS and RFS rates were 77.7% and 59.9%. Descriptive survival statistics and Kaplan–Meier analysis suggested that tumor size and vascular invasion had prognostic significance within this selected cohort. Tumor size >2.5 cm was associated with lower 5-year OS and RFS rates as compared with tumor size ≤2.5 cm (OS: 67.3% vs. 87.0%, hazard ratio [HR] = 2.431, 95% confidence interval [CI] = 1.443–4.093, *P* = 0.014; RFS: 50.3% vs. 67.6%, HR = 1.710, 95% CI 1.211–2.415, *P* = 0.002) (Table 2; Fig. 1a, b). Similarly, the 5-year OS and RFS rates were higher in patients without vascular invasion than in patients with vascular invasion (OS: 83.2% vs. 59.2%, HR = 3.033, 95% CI 1.827–5.035, *P* = 0.001; RFS: 63.7% vs. 47.2%, HR = 1.790, 95% CI 1.236–2.594, *P* = 0.002) (Table 2; Fig. 1c, d). Additionally, tumor grade was significantly associated with RFS (*P* = 0.003), but not with OS (*P* = 0.172) (Table 2; Fig. 1e, f).

**Table 2:** Univariate analysis of different prognostic factors for survival in 335 patients with sHCC (Cox proportional hazards regression)

Variable	Overall survival		Recurrence-free survival	
	HR (95% CI)	*P* value	HR (95% CI)	*P* value
Gender (male vs. female)	0.825 (0.374–1.816)	0.825	0.663 (0.366–1.202)	0.176
Age (>48 vs. ≤48 years)	1.014 (0.615–1.672)	0.331	1.171 (0.829–1.654)	0.371
ALT (≤40 vs. >40 U/L)	1.337 (0.812–2.201)	0.949	1.272 (0.902–1.795)	0.170
AFP (≤20 vs. >20 ng/mL)	1.230 (0.734–2.059)	0.130	0.912 (0.644–1.289)	0.601
Tumor size (≤2.5 vs. > 2.5 cm)	2.431 (1.443–4.093)	0.014	1.710 (1.211–2.415)	0.002
Tumor differentiation (well-moderate vs. poor-undifferentiated)	1.215 (0.679–2.175)	0.172	1.774 (1.221–2.578)	0.003
Vascular invasion (absent vs. present)	3.033 (1.827–5.035)	0.001	1.790 (1.236–2.594)	0.002
Envelope (absent vs. present)	0.920 (0.544–1.559)	0. 110	0.958 (0.668–1.375)	0. 816
Liver cirrhosis (absent vs. present)	1.516 (0.921–2.495)	0.496	1.329 (0.939–1.881)	0.108

*AFP* alpha-fetoprotein, *ALT* alanine aminotranferease, *HR* hazards ratio, *CI* confidence interval

### Multivariate Cox regression analysis

The clinicopathologic variables were examined using multivariate Cox regression analysis. The multivariate analysis confirmed that tumor size >2.5 cm (HR = 2.367, 95% CI 1.406–3.985, *P* = 0.001) and vascular invasion (HR = 2.954, 95% CI 1.781–4.900, *P* < 0.001) were independent prognostic factors for short OS in patients with sHCC (Table 3). Similarly, tumor size >2.5 cm (HR = 1.779, 95% CI 1.259–2.514, *P* = 0.001) and vascular invasion (HR = 1.699, 95% CI 1.165–2.477, *P* = 0.006) were shown to be independent and powerful predictors for RFS (Table 3).

**Table 3:** Multivariate analysis of different prognostic factors for survival in 335 patients with sHCC (Cox proportional hazards regression)

Variable	Overall survival		Recurrence-free survival	
	HR (95% CI)	*P* value	HR (95% CI)	*P* value
Tumor size (≤2.5 vs. >2.5 cm)	2.367 (1.406–3.985)	0.001	1.779 (1.259–2.514)	0.001
Tumor differentiation (well-moderate vs. poor-undifferentiated)	NA	NA	1.678 (1.148–2.453)	0.008
Vascular invasion (absent vs. present)	2.954 (1.781–4.900)	<0.001	1.699 (1.165–2.477)	0.006

*HR* hazards ratio, *CI* confidence interval, *NA* not applicable

### New prognostic model of tumor size and vascular invasion in sHCC

**Fig. 2:**
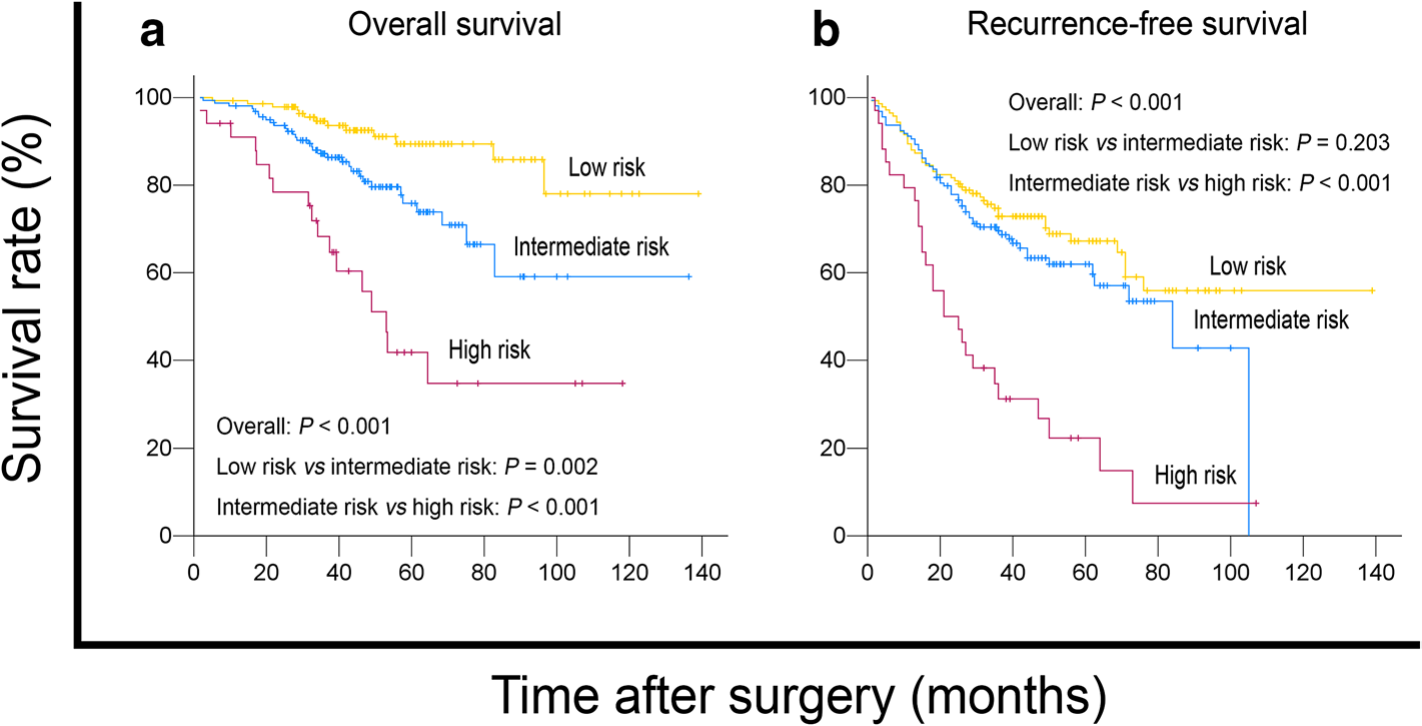
The proposed prognostic model successfully stratified risk for survival prediction of patients with sHCC (log-rank test). Using this model, these sHCC patients were stratified into three groups: low risk, *n* = 142; intermediate risk, *n* = 159; high risk, *n* = 34. **a** The OS curves of the three groups were significantly different (*P* < 0.001). **b** The RFS curves of the three groups were significantly different (*P* < 0.001)

Based on the results of our univariate and multivariate analyses, we proposed a new clinicopathologic prognostic model with two prognostic factors: tumor size and vascular invasion. We therefore designated a high-risk group with the presence of both factors (tumor size >2.5 cm and presence of vascular invasion), an intermediate-risk group with the presence of either factor (regardless of their identity), and a low-risk group with neither factor. This model successfully stratified risk (low, intermediate, and high) for OS and RFS prediction (both *P* < 0.001, Fig. 2).

## Discussion

In the present study, we have investigated the prognostic predictors in a large cohort of patients from an area of high prevalence of HBV infection and who underwent liver resection for sHCC. Importantly, tumor size and vascular invasion were independent prognostic factors determining the outcome of these patients, and a prognostic scoring model derived according to these two variables was shown to accurately predict survival in patients with sHCC.

Tumor size is a well-known risk factor for survival after hepatectomy for HCC [17–19]. However, the appropriate tumor size cutoff for prognosis predicting has been debated at length. Several studies have indicated that a tumor size >5 cm is of prognostic significance only in patients with multifocal tumors [19]. In contrast, Minagawa et al. [17] showed a significant difference in survival between patients with tumors <2 cm and those with tumors measuring 2–5 cm, irrespective of multifocality or vascular invasion. Moreover, Zhou et al. [20] reported that tumor size significantly associated with decreased OS; the 5-year OS rates for patients with sHCC tumors measuring ≤2.0, 2.1–3.0, and 3.1–5.0 cm were 82.5%, 66.3%, and 61.2%, respectively. Similarly, we found that tumor size >2.5 cm was associated with a short OS or RFS, even within a cohort of patients with tumors ≤3 cm. However, Nanashima et al. [21] reported no significant difference in OS between patients with solitary sHCC tumors <2 cm and those with tumors measuring 2–3 cm in size. Lu et al. [22] observed no significant difference in OS and RFS between patients with tumors measuring 1.1–2.0 and 2.1–3.0 cm. Taken together, differences in the clinicopathologic characteristics among cohorts, geographic backgrounds, tumor size cutoff, patient heterogeneity, small sample sizes, and different definitions of end points (OS or RFS) may contribute to the conflicting results. In our study, the findings of a significant association between tumor size and clinical outcome in sHCC patients may be strengthened by the consistent resection procedure and large sample size. Moreover, the rate of liver cirrhosis was 40% in the present study, which is much lower than that observed in previous studies of Chinese populations [23, 24]. Only patients with sHCC (≤3 cm) were included in our study, and this group is generally considered to have early stage HCC. This is one potential explanation for the low incidence of liver cirrhosis observed in the present study.

HCC is characterized by a propensity for vascular invasion [23], and vascular invasion is a clinicopathologic feature related to aggressive biological behavior of HCC. Over the past several decades, multiple studies have shown that vascular invasion is a strong predictor of outcome following hepatic resection and liver transplantation for HCC. Nevertheless, the relative prognostic importance of vascular invasion in sHCC remains controversial, and there is significant inter-study and intra-study variability in assessing vascular invasion. Shindoh et al. [25] reported that vascular invasion did not affect survival of patient with sHCC (≤2 cm), whereas others maintain the opposing view that vascular invasion is an independent prognostic factor for sHCC (≤3 cm) [26]. Our data showed that vascular invasion had an adverse effect on long-term survival in patients with sHCC. The presence of vascular invasion was associated with a significant decrease in both 5-year OS and RFS rates. However, a crossover exists in RFS curves between patients with and without vascular invasion. We thought that the individual differences of cohorts and the different effects of vascular invasion on short-term and long-term survival would have contributed to this crossover. Based on a meta-analysis of our results as well as those of other studies, the presence of vascular invasion is associated with an adverse prognosis in HCC [27]. An international consensus on precisely what is meant by vascular invasion in HCC could provide a more consistent assessment and therefore a more reliable prediction of prognosis and a better understanding of the pathophysiology of HCC angioinvasion.

The reported OS rates for patients with HCC following resection varies widely in the literature, with 5-year OS rates ranging from 35% to 70% [28–30]. Patients with sHCC are generally thought to have a good outcome and are often considered a relatively homogeneous group. Data from our study revealed that even patients with early-stage HCC could be stratified into subgroups with distinct long-term prognoses. An improved understanding of the factors that affect outcome in this group of patients may impact choice of follow-up strategies and salvage therapy as well as guiding future studies [13]. In this study, we analyzed data from a large, population-based cohort of patients with pathologically proven sHCC. The apparent homogeneity of this cohort with respect to extent of disease (≤3 cm in size, solitary tumor, and no nodal involvement or metastases) and surgical resection was notable. Despite this, we found that the 5-year OS rate in this group ranged from 59.2% to 83.2%. In the current study, we found that the proposed prognostic model containing both tumor size and vascular invasion could reflect the aggressive phenotype of sHCC. Thus, this combined model may be a useful prognostic indicator for sHCC. There are also intense ongoing efforts to integrate biomarkers into established clinicopathologic models to further improve their predictive ability.

## Conclusions

Tumor size and vascular invasion are feasible and useful prognostic factors for solitary sHCC (≤3 cm). The proposed prognostic model, based on tumor size and vascular invasion, is informative to predict the survival in sHCC patients undergoing hepatectomy.

## References

[1] Jemal A, Siegel R, Ward E, Hao Y, Xu J, Murray T (2008). Cancer statistics, 2008. CA Cancer J Clin.

[2] Ince N, Wands JR (1999). The increasing incidence of hepatocellular carcinoma. N Eng J Med.

[3] El-Serag HB, Mason AC (1999). Rising incidence of hepatocellular carcinoma in the United States. N Eng J Med.

[4] Cabibbo G, Craxi A (2010). Epidemiology, risk factors and surveillance of hepatocellular carcinoma. Eur Rev Med Pharmacol Sci..

[5] Cai MY, Tong ZT, Zheng F, Liao YJ, Wang Y, Rao HL (2011). EZH2 protein: a promising immunomarker for the detection of hepatocellular carcinomas in liver needle biopsies. Gut.

[6] Llovet JM, Burroughs A, Bruix J (2003). Hepatocellular carcinoma. Lancet.

[7] An FQ, Matsuda M, Fujii H, Tang RF, Amemiya H, Dai YM (2001). Tumor heterogeneity in small hepatocellular carcinoma: analysis of tumor cell proliferation, expression and mutation of p53 AND beta-catenin. Int J Cancer.

[8] Yuen MF, Cheng CC, Lauder IJ, Lam SK, Ooi CG, Lai CL (2000). Early detection of hepatocellular carcinoma increases the chance of treatment: Hong Kong experience. Hepatology.

[9] Bruix J, Sherman M (2005). Practice Guidelines Committee AAftSoLD. Management of hepatocellular carcinoma. Hepatology.

[10] Yamamoto M, Takasaki K, Otsubo T, Katsuragawa H, Katagiri S, Yoshitoshi K (2004). Favorable surgical outcomes in patients with early hepatocellular carcinoma. Ann Surg.

[11] Villanueva A, Hoshida Y, Toffanin S, Lachenmayer A, Alsinet C, Savic R (2010). New strategies in hepatocellular carcinoma: genomic prognostic markers. Clin Cancer Res.

[12] Chan SL, Johnson PJ, Mo F, Berhane S, Teng M, Chan AW (2014). International validation of the Chinese university prognostic index for staging of hepatocellular carcinoma: a joint United Kingdom and Hong Kong study. Chin J cancer..

[13] Nathan H, Schulick RD, Choti MA, Pawlik TM (2009). Predictors of survival after resection of early hepatocellular carcinoma. Ann Surg.

[14] Ko CJ, Chien SY, Chou CT, Chen LS, Chen ML, Chen YL (2011). Factors affecting prognosis of small hepatocellular carcinoma in Taiwanese patients following hepatic resection. Can J Gastroenterol.

[15] Kong M, Hong SE (2015). Optimal follow-up duration for evaluating objective response to radiotherapy in patients with hepatocellular carcinoma: a retrospective study. Chin J Cancer..

[16] Edmondson HA, Steiner PE (1954). Primary carcinoma of the liver: a study of 100 cases among 48,900 necropsies. Cancer.

[17] Minagawa M, Ikai I, Matsuyama Y, Yamaoka Y, Makuuchi M (2007). Staging of hepatocellular carcinoma: assessment of the Japanese TNM and AJCC/UICC TNM systems in a cohort of 13,772 patients in Japan. Ann Surg.

[18] The Liver Cancer Study Group of Japan (1994). Predictive factors for long term prognosis after partial hepatectomy for patients with hepatocellular carcinoma in Japan. Cancer.

[19] Vauthey JN, Lauwers GY, Esnaola NF, Do KA, Belghiti J, Mirza N (2002). Simplified staging for hepatocellular carcinoma. J Clin Oncol.

[20] Zhou XD, Tang ZY, Yang BH, Lin ZY, Ma ZC, Ye SL (2001). Experience of 1000 patients who underwent hepatectomy for small hepatocellular carcinoma. Cancer.

[21] Nanashima A, Tobinaga S, Masuda J, Miyaaki H, Taura N, Takeshita H (2010). Selecting treatment for hepatocellular carcinoma based on the results of hepatic resection and local ablation therapy. J Surg Oncol.

[22] Lu XY, Xi T, Lau WY, Dong H, Xian ZH, Yu H (2011). Pathobiological features of small hepatocellular carcinoma: correlation between tumor size and biological behavior. J Cancer Res Clin Oncol.

[23] Du M, Chen L, Zhao J, Tian F, Zeng H, Tan Y (2014). Microvascular invasion (MVI) is a poorer prognostic predictor for small hepatocellular carcinoma. BMC Cancer.

[24] Wu FS, Zhao WH, Liang TB, Ma ZM, Teng LS, Wang M (2005). Survival factors after resection of small hepatocellular carcinoma. Hepatobiliary Pancreat Dis Intern.

[25] Shindoh J, Andreou A, Aloia TA, Zimmitti G, Lauwers GY, Laurent A (2013). Microvascular invasion does not predict long-term survival in hepatocellular carcinoma up to 2 cm: reappraisal of the staging system for solitary tumors. Ann Surg Oncol.

[26] Zhou YM, Yang JM, Li B, Yin ZF, Xu F, Wang B (2010). Risk factors for early recurrence of small hepatocellular carcinoma after curative resection. Hepatobiliary Pancreat Dis Intern.

[27] Rodriguez-Peralvarez M, Luong TV, Andreana L, Meyer T, Dhillon AP, Burroughs AK (2013). A systematic review of microvascular invasion in hepatocellular carcinoma: diagnostic and prognostic variability. Ann Sur Oncol..

[28] Bruix J, Llovet JM (2002). Prognostic prediction and treatment strategy in hepatocellular carcinoma. Hepatology.

[29] Fuster J, Garcia-Valdecasas JC, Grande L, Tabet J, Bruix J, Anglada T (1996). Hepatocellular carcinoma and cirrhosis. Results of surgical treatment in a European series. Ann Sur..

[30] Franco D, Capussotti L, Smadja C, Bouzari H, Meakins J, Kemeny F (1990). Resection of hepatocellular carcinomas. Results in 72 European patients with cirrhosis. Gastroenterology.

